# Syntheses and Biological Activity of Some Derivatives of C-9154 Antibiotic

**DOI:** 10.1155/2012/148235

**Published:** 2012-08-06

**Authors:** Isaac Asusheyi Bello, George Iloegbulam Ndukwe, Joseph Olorunju Amupitan, Rachael Gbekele Ayo, Francis Oluwole Shode

**Affiliations:** ^1^Department of Chemistry, Ahmadu Bello University, Zaria 810001, Nigeria; ^2^Division of Agricultural Colleges, College of Agriculture, Ahmadu Bello University, Zaria 810001, Nigeria; ^3^School of Chemistry and Biochemistry, University of Zululand, Empangeni 3880, South Africa

## Abstract

This research was undertaken to design several new antibiotics, by structurally modifying the C-9154 antibiotic, simultaneously improving its activity and lowering toxicity. This was achieved by synthesizing an analogue to the C-9154 antibiotic and seven derivatives of this analogue. The approach was to significantly reduce the polarity of the synthesized analogue in the derivatives to achieve increased permeability across cell membranes by conversion of the highly polar carboxylic group to an ester functional group. The compounds were fully characterized using infrared, GC-MS, and 1D and 2D NMR experiments. The *in vitro* biological activity of the compounds showed that the derivatives were more active than the analogue as was anticipated and both were more active than the standard drugs used for comparison. Work is ongoing to establish applications for the compounds as antiplasmodials, antivirals, anticancers/tumours, antitrypanosomiasis, anthelminthic, and as general antibiotics for human, veterinary, and even agricultural use as they had marked effect on both Gram-positive and Gram-negative bacteria and some fungi.

## 1. Introduction

Vuillemin (1889), a French bacteriologist, was first to suggest using the word “antibiosis,” (against life) to describe the group of compounds that exhibited biological action against microorganisms [1]. Selman Waksman, the discoverer of streptomycin, later changed this term to antibiotic in 1942 [2]. The term “antibiotic” according to Waksman is used to describe any substance produced by a microorganism that is detrimental to the growth of other microorganisms in high dilution or at low concentration [3]. With current advances in medicinal chemistry, antibiotics are mostly semisynthetic modifications of various natural compounds [4]. These include, for example, the beta-lactam antibiotics, like the penicillins, the cephalosporins, and the carbapenems. Some antibiotic compounds are still being isolated from natural sources, for example, the aminoglycosides, whereas other antibiotics like the sulfonamides, the quinolones, and the oxazolidinones are produced entirely by chemical synthesis [4]. This implies that synthesis of antibiotic compounds plays an important and vital role in the fight against disease-causing organisms.

The problem of resistance to antibiotics on the part of the microorganism, the adverse side effects associated with antibiotics in current use, and the difficulty in obtaining these antibiotics in large (commercial) quantities from their natural sources imply that newer antibiotics have to be constantly sought to address these problems to give man the needed advantage in the ongoing battle between microbes and men. Synthesis of previously characterized antibiotics with structural modifications to imbue desirable qualities or remove undesirable ones provides a way to assist man in this great battle.

During studies on screening for antibiotics that showed activity against bacteria resistant to various known antibiotics, a new antibiotic with a broad antibacterial spectrum was isolated from the whole agar culture of a *Streptomyces* strain, NR-7GGI. This *Streptomyces* species was called *Streptomyces kurssanovii* and the isolated antibiotic referred to as fumaramidmycin [5]. Another researcher working independently and slightly earlier than the previous researcher also found that a new species of *Streptomyces*, *Streptomyces ishigakiensis*, produced a novel antibiotic which was named C-9154 [6]. The two new antibiotics were found from structural studies to be the same compound [6, 7]. This new antibiotic was found to inhibit the growths of various microorganism at concentrations between 10–>100 *μ*g/mL [6]. It was also shown to be active against certain strains that were resistant to ampicillin, cephalosporin, chloramphenicol, gentamicin, kanamycin, macrolides, neomycin, sulfonamides, streptomycin, and tetracyclines at concentrations between 3.12–>200 *μ*g/mL [5]. Its intraperitoneal LD_50_ value in mice was found to be 75–100 mg/kg while its oral LD_50_ was found to be 1.25–2.5 g/kg [5].

**Figure 1:**
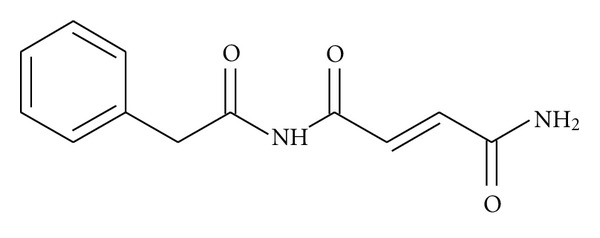
C-9154 antibiotic.

The structure of C-9154 (Figure 1) was determined using elemental analysis procedures, IR and UV measurements and NMR and GC-MS experiments [6, 7].

Analysis of the structure of C-9154 antibiotic showed that it was made up of two fragments, namely, phenylacetic acid and fumaramide [6, 7]. Analogues of the C-9154 antibiotic have been previously synthesized [7–10]. These analogues were shown to have antibacterial activity against *Staphylococcus aureus *and* Escherichia coli* ranging from 15 *μ*g/mL [10] to 4000 *μ*g/mL [9]. We now report the synthesis of an analogue and seven derivatives of the C-9154 antibiotic and their biological activity.

## 2. Materials and Method

Infrared spectra were determined using a PerkinElmer Spectrum 100 series Universal ATR. 1D and 2D NMR experiments were carried out using a Bruker av400 MHz NMR. The gc/ms spectra were taken using an Agilent Technologies 6890 series GC coupled with an Agilent 5973 Mass Selective detector. All weight measurements were taken using a Precisa 3100C top loading balance. Melting points were determined using a Micro Melting point apparatus (Reichart Austria), uncorrected. All chemicals and reagents unless otherwise stated were obtained from Merck Chemicals, Germany.

### 2.1. Synthesis of C-9154 Analogue

**Figure 2:**
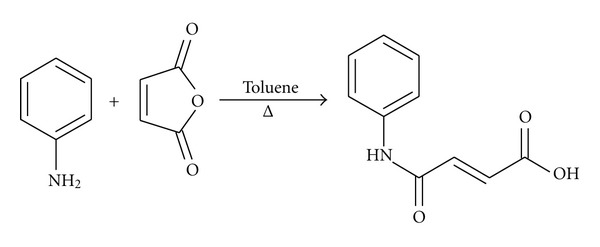
Reaction scheme 1.

N-phenyl fumaramic acid was prepared according to reaction scheme 1 (Figure 2). Aniline (1.0 g, 11.0 mmol) in toluene (5 mL) was transferred to a round bottom flask containing maleic anhydride (1.3 g, 13.3 mmol) in toluene (5 mL). The mixture was refluxed at 100°C with continuous stirring for 2 hrs. The reaction was allowed to cool to room temperature and vacuum filtered using a Buchner funnel. The residue was washed using ethyl acetate and dried to afford a grey solid labeled IA/01/1 (1.9 g, 90.0%). TLC was used to determine that the reaction had gone to completion. IR, 1D and 2D NMR, and GC-MS were used to identify the compound as the desired N-phenyl fumaramic acid. Its melting point was determined to be 195-196°C.

### 2.2. Synthesis of Derivatives of C-9154 Analogue

**Figure 3:**
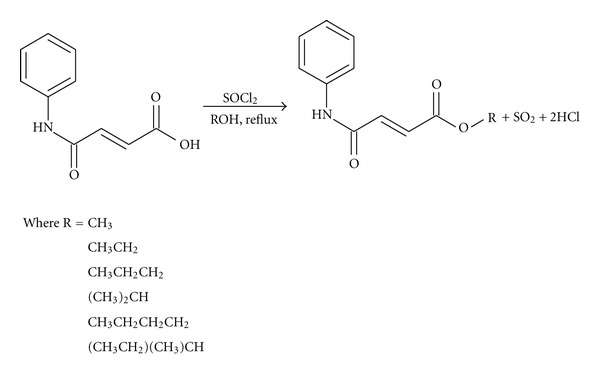
Reaction scheme 2.

This analogue was then converted to its ester derivatives using methanol, ethanol, n-propanol, isopropanol, n-butanol, 2-butanol, and t-butanol, respectively, and thionyl chloride (SOCl_2_) as catalyst according to reaction scheme 2 (Figure 3).

Seven portions of IA/01/1 (0.5 g, 2.6 mmol) were individually transferred to seven round bottomed flasks each containing thionyl chloride (2 mL) in an ice bath. The excess thionyl chloride was removed using a rotary evaporator. Methanol (10 mL), ethanol (10 mL), n-propanol (10 mL), isopropanol (10 mL), n-butanol (10 mL), 2-butanol (10 mL), and t-butanol (10 mL) were respectively added to each flask and the mixtures refluxed for 10 minutes. At the end of the reactions as determined by TLC, saturated sodium carbonate (Na_2_CO_3_) solution was added to each flask until the solutions just turned alkaline as indicated by litmus paper. Water (20 mL) was added to each flask and the mixtures were transferred to individual separatory funnels. The mixtures were each extracted using dichloromethane (2 × 25 mL). The combined dichloromethane fractions were dried using anhydrous sodium sulphate (Na_2_SO_4_) and concentrated to give light yellow to clear oils. These were chromatographed on individual silica gel columns and eluted using ethyl acetate : hexane (3 : 7), to give the desired esters which crystallized on standing. All the esters except the 2-butyl esters were obtained as crystalline solids (Table 1). IR, 1D and 2D NMR, and GC-MS were used to identify the compounds as the desired esters.

### 2.3. Biological Screening

The analogue and its derivatives were successfully synthesized and characterized. These compounds were then subjected to biological screening *in vitro* to ascertain their activity and the concentration at which this activity was exhibited.

This was carried out using zones of inhibition measurements, minimum inhibitory concentration measurements, and minimum bactericidal/fungicidal concentration measurements. Fourteen (14) microorganisms were selected for the screening to include both gram-positive and gram-negative bacteria and some fungi. These microorganisms are: Methicillin-Resistant *Staphylococcus aureus, Staphylococcus aureus, Streptococcus pyogenes, Bacillus subtilis, *and *Corynebacterium ulcerans* for Gram-positive bacteria*, Escherichia coli, Proteus mirabilis, Pseudomonas aeruginosa, Salmonella typhii, Shigella dysenteriae, *and *Klebsiella pneumonia* for Gram-negative bacteria, and* Candida albicans, Aspergillus nigre*, and *Trichophyton rubrum* for fungi.

### 2.4. Zones of Inhibition

The antimicrobial activity (Table 2) of the synthesized analogue and derivatives was determined using some pathogenic microorganisms obtained from the Department of Medical Microbiology, Ahmadu Bello University Teaching Hospital, Zaria, Nigeria. All isolates were checked for purity and maintained in slants of blood agar.

The analogue (0.1 mg) and the derivatives (0.05 mg) were each weighed and dissolved in DMSO (10 mL) to obtain a concentration of 10 *μ*g/mL and 5 *μ*g/mL, respectively. This was the initial concentration used to check the antimicrobial activities of the compounds.

Mueller Hinton, (Oxoid, England) and Sabouraud agar, (Oxoid, England) were the growth media used for the bacteria and fungi, respectively. The media were prepared according to the manufacturer's instructions, sterilized at 121°C for 15 minutes and were poured into sterile Petri dishes. The plates were allowed to cool and solidify. Diffusion method was the method used for screening the compounds. The sterilized media were seeded with a standard inoculum (0.1 mL) of the test microorganisms. This was spread evenly over the surface of the plate by using a sterile swab. The plates were dried at 37°C for 30 minutes. By the use of a standard cork-borer of 6 mm in diameter, a well was cut at the centre of each seeded plate. 0.1 mL of the compounds was then introduced into the well. The plates were then incubated at 37°C for 24 hrs for the bacteria and 30°C for 48 hrs for the fungi, after which the plates were observed for zones of inhibition of growth. The zones were measured using a pair of dividers and a ruler and the result recorded in millimeters. 

The activity of the compounds was compared against two standard drugs; Sparfloxacin (antibacterial), and Fluconazole (antifungal) which were subjected to the same treatment as the synthesized compounds.

### 2.5. Minimum Inhibitory Concentration

The minimum inhibitory concentrations (Table 3) of the compounds were carried out using broth dilution method. Mueller Hinton, (Oxoid, England) and Sabouraud dextrose broth, (Oxoid, England) were prepared according to the manufacturer's instructions, and 10 mL was dispensed into test tubes and the broths were sterilized at 121°C for 15 minutes, the broths were allowed to cool. 

McFarland's turbidity scale number 0.5 was prepared to give turbid solution. Normal saline was prepared, and the test microorganisms were inoculated and incubated at 37°C for 6 hrs. Dilution of the test microorganisms were done continuously in the normal saline until the turbidity matched that of the McFarland's scale by visual comparison. At that point the test microbe was at a concentration of 1.5 × 10^8^ cfu/mL. Two-fold serial dilutions of the compounds in the broth were made to obtain the different concentrations of the compounds in the broth. Having obtained the different concentrations, 0.1 mL of the standard inoculum of the test microorganisms in the normal saline was then inoculated into the different concentrations, and then incubated at 37°C for 24 hrs for the bacteria and 30°C for 48 hrs for the fungi, after which each test tube was observed for turbidity (growth). The MIC was the test tube with the lowest concentration of the compounds which showed no turbidity.

### 2.6. Minimum Bactericidal/Fungicidal Concentration

MBC (Table 3) was carried out to check whether test microorganisms were killed or only their growths were inhibited. Mueller Hinton, (Oxoid, England) and Sabouraud dextrose agar, (Oxoid, England) were prepared, sterilized, and poured into sterile Petri dishes. They were allowed to cool and solidify. The content of the MIC in the serial dilution were then subcultured onto the prepared media. They were then incubated at 37°C for 24 hrs for the bacteria and 30°C for 48 hrs for the fungi after which each plate was observed for colony growth. The MBC/MFC was the plate with lowest concentration of the compounds without colony growth.

## 3. Results and Discussion

A total of eight compounds were synthesized and fully characterized using 1D and 2D NMR experiments, infrared spectrophotometry, and gas chromatography-mass spectrometry.

The analogue was synthesized by reaction between the required N-phenyl amine (aniline) and maleic anhydride to get the desired fumaramic acid. This analogue was then converted to its methyl, ethyl, n-propyl, isopropyl, n-butyl, 2-butyl, and t-butyl esters using the Fischer-Speier esterification procedure. The results are presented below.

### 3.1. IA/01/1 (N-Phenylamino Fumaramic Acid)

 Grey powdery solid; melting point, 195-196°C.

^13^C-NMR (400 MHz, CDCl_3_ and DMSO-d_6_). 165.1 (C10), 163.7 (C7), 136.6 (C1), 133.7 (C9), 132.7 (C8), 128.4 (C3 and C5), 125.0 (C4), 120.3 (C2 and C6).

^1^H-NMR (400 MHz, CDCl_3_ and DMSO-d_6_): *δ* 6.23 (1H, d, *J* = 12.69 Hz, H-9), 6.55 (1H, d, *J* = 12.73 Hz, H-8), 7.09 (1H, t, *J* = 7.40 Hz, H-4), 7.27 (2H, *t*, *J* = 7.88 Hz, H-3 and H-5), 7.57 (2H, *d*, *J* = 7.84 Hz, H2 and H-6), 7.68 (1H, s, 1-NH), 10.79 (1H, s, 10-OH). EI-MS: *m*/*z* 173 {[*M*−*H*]^+^, 100%}. IR_vmax_ (neat) cm^−1 ^: 3271.36 (N-H), 3071.75 (O-H), 2883.57 (C-H), 1694.60, 1618.99 (C=O).

### 3.2. IA/24/1/B (Methyl N-Phenylamino Fumaramate)

Bright yellow crystalline solid; melting point, 74°C.

^13^C-NMR (400 MHz, CDCl_3_). 167.4 (C7), 161.5 (C10), 137.8 (C1), 140.5 (C9), 124.9 (C8), 129.0 (C3 and C5), 124.6 (C4), 120.1 (C2 and C6), 52.8 (C11).

^1^H-NMR (400 MHz, CDCl_3_) *δ* 3.83 (3H, s, H-11), 6.21 (1H, d, *J* = 13.33 Hz, H-8), 6.42 (1H, d, *J* = 13.25 Hz, H-9), 7.11 (1.0H, *t*, *J* = 7.38 Hz, 4-H), 7.32 (2H, *t*, *J* = 7.60 Hz, H-3 and H-5), 7.64 (2H, d, *J* = 7.88 Hz, H-2 and H-6), 10.82 (1H, s, 1-NH). EI-MS: *m*/*z* 205 {[M]^+^, 50%}. IR_vmax_ (neat) cm^−1 ^: 3283.23 (N-H), 3079.91, 2953.75 (C-H), 1730.94, 1666.54 (C=O), 1206.53, 1165.92 (C-O).

### 3.3. IA/21/1/B (Ethyl N-Phenylamino Fumaramate)

 Light brown crystalline solid; melting point, 63°C.

^13^C-NMR (400 MHz, CDCl_3_). 166.7 (C7), 162.0 (C10), 137.9 (C1), 138.9 (C9), 126.0 (C8), 129.0 (C3 and C5), 124.6 (C4), 120.1 (C2 and C6), 61.9 (C11), 14.0 (C12).

^1^H-NMR (400 MHz, CDCl_3_) *δ* 1.30 (3H, t, *J* = 7.20 Hz, H-12), 4.26 (2H, *q*, *J* = 7.14 Hz, H-11), 6.16 (1H, *d*, *J* = 13.09 Hz, H-8), 6.39 (1H, *d*, *J* = 13.09 Hz, H-9), 7.10 (1H, *t*, *J* = 7.36 Hz, H-4), 7.31 (2H, *t*, *J* = 7.84 Hz, H-3 and H-5), 7.65 (2H, *d*, *J* = 7.96 Hz, H-2 and H-6), 10.7 (1H, s, 1-NH). EI-MS: *m*/*z* 219 {[M]^+^, 50%}. IR_vmax_ (neat) cm^−1 ^: 3285.55 (N-H), 3063.98, 2926.30 (C-H), 1716.89, 1667.67 (C=O), 1176.46, 1166.06 (C-O).

### 3.4. IA/22/1/B (n-Propyl N-Phenylamino Fumaramate)

Dark brown crystalline solid; melting point, 55°C.

^13^C-NMR (400 MHz, CDCl_3_). 166.7 (C7), 162.5 (C10), 137.9 (C1), 137.2 (C9), 126.6 (C8), 128.9 (C3 and C5), 124.5 (C4), 120.2 (C2 and C6), 67.3 (C11), 21.7 (C12) 10.3 (C13).

^1^H-NMR (400 MHz, CDCl_3_) *δ* 0.83 (3H, t, *J* = 7.42 Hz, H-13), 1.57 (2H, m, H-12), 4.04 (2H, t, *J* = 6.74 Hz), 6.04 (1H, d, *J* = 12.69 Hz, H-8), 6.31 (1H, d, *J* = 12.69 Hz, H-9), 7.00 (1H, t, *J* = 7.42 Hz, H-4), 7.21 (2H, d, *J* = 7.64 Hz, H-3 and H-5), 7.56 (2H, d, *J* = 7.76 Hz, H-2 and H-6), 10.3 (1H, s, 1-NH). EI-MS: *m*/*z* 233 {[M]^+^, 50%}. IR_vmax_ (neat) cm^−1 ^: 3259.72 (N-H), 3092.97, 2878.49 (C-H), 1719.46, 1662.54 (C=O), 1205.45, 1185.79 (C-O).

### 3.5. IA/23/1/B (Isopropyl N-Phenylamino Fumaramate)

Dark brown crystalline solid; melting point, 70°C.

^13^C-NMR (400 MHz, CDCl_3_). 166.4 (C7), 161.6 (C10), 137.9 (C1), 140.3 (C9), 125.8 (C8), 129.0 (C3 and C5), 124.5 (C4), 120.1 (C2 and C6), 70.0 (C11), 21.7 (C12 and C13).

^1^H-NMR (400 MHz, CDCl_3_) *δ* 1.32 (6H, d, *J* = 6.28 Hz, H-12 and H-13), 5.15 (1H, m, H-11), 6.18 (1H, d, *J* = 13.36 Hz, H-8), 6.41 (1H, d, *J* = 13.45 Hz, H-9), 7.12 (1H, t, *J* = 7.38 Hz, H-4), 7.34 (2.1H, d, *J* = 15.81 Hz, H-3 and H-5), 7.67 (1.9H, d, *J* = 7.84 Hz, H-2 and H-6), 11.0 (1H, s, 1-NH). EI-MS: *m*/*z* 233 {[M]^+^, 50%}. IR_vmax_ (neat) cm^−1 ^: 3238.13 (N-H), 3074.64, 2936.22 (C-H), 1729.86, 1660.48 (C=O), 1166.93, 1103.15 (C-O).

### 3.6. IA/19/1/B (n-Butyl N-Phenylamino Fumaramate)

 Yellow waxy solid; melting point, 42°C.

^13^C-NMR (400 MHz, CDCl_3_). 166.6 (C7), 162.8 (C10), 136.2 (C1), 138.0 (C9), 126.8 (C8), 128.8 (C3 and C5), 124.5 (C4), 120.2 (C2 and C6), 65.4 (C11), 30.3 (C12), 19.0 (C13), 13.6 (C14).

^1^H-NMR (400 MHz, CDCl_3_) *δ* 0.84 (3H, t, *J* = 7.60 Hz, H-14), 1.31 (2H, m, H-13), 1.56 (2H, m, H-12), 4.12 (2H, t, *J* = 6.78 Hz, H-11), 6.07 (1H, d, *J* = 12.69 Hz, H-8), 6.38 (1H, d, *J* = 12.53 Hz, H-9), 7.04 (1H, t, *J* = 7.40 Hz, H-4), 7.24 (2H, t, *J* = 7.64 Hz, H-3 and H-5), 7.63 (2H, d, *J* = 8.20 Hz, H-2 and H-6), 10.2 (1H, s, 1-NH). EI-MS: *m*/*z* 247 {[M]^+^, 75%}. IR_vmax_ (neat) cm^−1 ^: 3298.98 (N-H), 2957.43, 2872.97 (C-H), 1720.16, 1659.63 (C=O), 1223.16, 1178.85 (C-O).

### 3.7. IA/20/1/B (2-Butyl N-Phenylamino Fumaramate)

 Golden brown oil.

^13^C-NMR (400 MHz, CDCl_3_). 166.4 (C7), 162.0 (C10), 138.0 (C1), 139.0 (C9), 126.3 (C8), 129.0 (C3 and C5), 124.5 (C4), 120.1 (C2 and C6), 74.4 (C11), 28.6 (C12), 19.2 (C14), 9.6 (C13).

^1^H-NMR (400 MHz, CDCl_3_) *δ* 0.87 (3H, t, *J* = 7.46 Hz, H-13), 1.23 (3H, d, *J* = 6.20 Hz, H-14), 1.58 (2H, m, H-12), 4.92 (1H, m, H-11), 6.11 (1H, d, *J* = 13.13 Hz, H-8), 6.35 (1H, d, *J* = 13.13 Hz, H-9), 7.06 (1H, t,  *J* = 7.36 Hz), 7.27 (2H, d, *J* = 15.81 Hz), 7.62 (2H, d, *J* = 7.68 Hz), 10.8 (1H, s, 1-NH). EI-MS: *m*/*z* 247 {[M]^+^, 50%}. IR_vmax_ (neat) cm^−1 ^: 3329.95 (N-H), 2972.12, 2878.77 (C-H), 1709.62, 1663.31 (C=O), 1245.78, 1175.08 (C-O).

The results show that the synthesized antibiotics had remarkable activity in the range 0.625–10 *μ*g/mL against the microorganisms for which they were active against. 

The derivatives showed higher activity than the analogue as was anticipated. This could be due to the reduction in polarity when the highly polar carboxylic functional group was converted to the less polar ester functional group. This has been shown to increase permeability across cell membranes [11–13]. All the synthesized compounds showed better activity than the standard drugs used for comparison. The antibacterial standard drug, sparfloxacin, was up to four-fold less active than most of the synthesized derivatives while the antifungal standard drug, fluconazole, was up to ten-fold less active than the synthesized analogue and derivatives for the microorganism against which they were active. All the synthesized compounds except (IA/01/1) and (IA/19/1/B) were able to inhibit the growth of *C. ulcerans* whereas the standard antibacterial could not. All the derivatives and the standard antifungal could not inhibit the growth of *A. nigre*, but the analogue (IA/01/1) was able to inhibit its growth. Two of the synthesized compounds (IA/19/1/B) and (IA/24/1/B) could also not inhibit the growth of *S. pyogenes*. All the synthesized compounds could not inhibit the growths of *P. aeruginosa* and *T. rubrum*. 

## 4. Conclusion

The results show that the synthesized compounds have a clear advantage over the tested standard drugs, and this has opened up the possibility of their application in the treatment of various ailments for which the tested microorganism are responsible for. The possible applications of these compounds are endless and with further studies could compliment or even replace some of the antibiotic drugs currently in the market if they are found to be less toxic and better tolerated. Other applications for the new antibiotics are in the veterinary and agricultural fields where they could be useful in combatting some of the diseases that plague both animals and plants.

Some *in vivo* work is being carried out to establish the activity of the synthesized compounds as anticancer, anti-HIV, antimalarial, antitrypanosomiasis, antituberculosis, and so forth.

## Figures and Tables

**Table 1:** Synthesized C-9154 analogue and its derivatives.

Sample code	Type	Yield (mg)	Melting point (°C)	Physical state
IA/01/1	C-9154 analogue	90%	195-196	White powdery solid
IA/24/1/B	Methyl ester	160, (0.78 mmol)	74	Bright yellow crystalline solid
IA/21/1/B	Ethyl ester	100, (0.46 mmol)	63	Light brown crystalline solid
IA/22/1/B	n-propyl ester	170, (0.73 mmol)	55	Dark brown crystalline solid
IA/23/1/B	Isopropyl ester	105, (0.45 mmol)	70	Dark brown crystalline solid
IA/19/1/B	n-butyl ester	200, (0.81 mmol)	42	Yellow waxy solid
IA/20/1/B	2-butyl ester	150, (0.61 mmol)	40	Golden brown oil
IA/25/1/B	t-butyl ester	2, (~0.01 mmol)	Not determined	

The yield from the t-butyl derivative was too little and could not be optimized. This was probably due to steric hindrance from the methyl groups.

**Table 2:** Zones of inhibition (mm) of the analogue and derivatives.

	IA/01/1	IA/24/1/B	IA/21/1/B	IA/22/1/B	IA/23/1/B	IA/19/1/B	IA/20/1/B	DMSO	Sparfloxacin	Fluconazole
	(10 *μ*g/mL)	(5 *μ*g/mL)	(5 *μ*g/mL)	(5 *μ*g/mL)	(5 *μ*g/mL)	(5 *μ*g/mL)	(5 *μ*g/mL)		(20 *μ*g/mL)	(50 *μ*g/mL)
MRSA	20	32	29	35	32	34	31	0	22	0
*S. aureus*	28	30	32	30	32	31	32	0	27	0
*S. pyogenes*	24	0	30	37	36	0	29	0	24	0
*B. subtilis*	30	26	32	32	30	29	31	0	30	0
*C. ulcerans*	0	30	29	27	30	0	27	0	0	0
*E. coli*	27	27	29	22	25	29	27	0	27	0
*P. mirabilis*	22	25	27	27	24	31	26	0	22	0
*P. aeruginosa*	0	0	0	0	0	0	0	0	20	0
*S. typhii*	26	30	27	29	31	29	22	0	21	0
*S. dysenteriae*	24	27	27	35	32	31	24	0	27	0
*K. pneumoniae*	27	24	27	30	31	29	27	0	25	0
*C. albicans*	22	22	24	27	30	24	21	0	0	24
*A. nigre*	21	0	0	0	0	0	0	0	0	0
*T. rubrum*	0	0	0	0	0	0	0	0	0	20

**Table 3:** Minimum inhibitory concentration (above) and minimum bactericidal/fungicidal concentration (below) of the analogue and derivatives (*μ*g/mL).

	IA/01/1	IA/24/1/B	IA/21/1/B	IA/22/1/B	IA/23/1/B	IA/19/1/B	IA/20/1/B	DMSO	Sparfloxacin	Fluconazole
MRSA	2.5	0.625	1.25	0.625	0.625	0.625	0.625	ND	10	ND
	10	2.5	2.5	1.25	2.5	1.25	2.5			

*S. aureus*	2.5	0.625	0.625	0.625	0.625	0.625	0.625	ND	10	ND
	5	2.5	2.5	2.5	2.5	2.5	2.5			

*S. pyogenes*	2.5	ND	0.625	0.625	0.625	ND	1.25	ND	10	ND
	10	ND	2.5	1.25	1.25	ND	2.5			

*B. subtilis*	1.25	1.25	0.625	0.625	0.625	1.25	0.625	ND	5	ND
	5	5	1.25	2.5	2.5	2.5	1.25			

*C. ulcerans*	ND	0.625	1.25	1.25	0.625	ND	1.25	ND	ND	ND
	ND	2.5	2.5	2.5	2.5	ND	5			

*E. coli*	2.5	1.25	1.25	1.25	1.25	1.25	1.25	ND	10	ND
	5	2.5	2.5	5	5	2.5	5			

*P. mirabilis*	2.5	1.25	1.25	1.25	1.25	0.625	1.25	ND	10	ND
	10	5	2.5	2.5	5	2.5	5			

*P. aeruginosa*	ND	ND	ND	ND	ND	ND	ND	ND	10	ND
	ND	ND	ND	ND	ND	ND	ND			

*S. typhii*	2.5	0.625	1.25	1.25	0.625	1.25	1.25	ND	10	ND
	5	2.5	5	2.5	2.5	2.5	5			

*S. dysenteriae*	2.5	1.25	1.25	0.625	0.625	0.625	1.25	ND	5	ND
	10	2.5	2.5	1.25	2.5	2.5	5			

*K. pneumoniae*	2.5	1.25	1.25	0.625	0.625	1.25	1.25	ND	5	ND
	5	5	5	2.5	2.5	2.5	2.5			

*C. albicans*	2.5	1.25	1.25	1.25	0.625	1.25	1.25	ND	ND	25
	10	5	5	2.5	2.5	5	5			

*A. nigre*	2.5	ND	ND	ND	ND	ND	ND	ND	ND	ND
	10	ND	ND	ND	ND	ND	ND			

*T. rubrum*	ND	ND	ND	ND	ND	ND	ND	ND	ND	25
	ND	ND	ND	ND	ND	ND	ND			

Upper values are MIC and lower values are MBC or MFC as the case may be. ND: not determined.
